# Life-Suspending Technologies, Cryonics, and Catastrophic Risks

**DOI:** 10.1007/s11948-024-00498-w

**Published:** 2024-08-09

**Authors:** Andrea Sauchelli

**Affiliations:** Faculty of Arts, Department of Philosophy & Hong Kong Catastrophic Risk Centre, Lingnan University, Castle Peak Road, Tuen Mun (New Territories), Hong Kong SAR, HSH213 China

**Keywords:** Intergenerational ethics, Catastrophic risks, Existential risks, Life-suspending technologies, Cryonics, Climate change, Future generations

## Abstract

I defend the claim that life-suspending technologies can constitute a catastrophic and existential security factor for risks structurally similar to those related to climate change. The gist of the argument is that, under certain conditions, life-suspending technologies such as cryonics can provide self-interested actors with incentives to efficiently tackle such risks—in particular, they provide reasons to overcome certain manifestations of generational egoism, a risk factor of several catastrophic and existential risks. Provided we have reasons to decrease catastrophic and existential risks such as climate change, we also have a (defeasible) reason for investing in developing and making life-suspending technologies (more) widespread.

Some argue that future generations are not represented in the decision-making processes of present generations and are thus disadvantaged and powerless. Future generations would be powerless with respect to present generations in the sense that their actions will not (causally) affect us, whereas what we do will affect them. For example, the Maltese delegation to the 1992 United Nations Conference on Environment and Development described the situation of future generations relative to the present generation as follows:

Future generations are inherently disadvantaged with respect to present generations in three important ways: (a) they are ‘downstream’ in time from us and thus subject to the long-term consequences of our actions; (b) they are ‘mute’, having no representatives among present generations and so their interests are often neglected in present socio-economic and political planning; and (c) they cannot plea[d] or bargain for reciprocal treatment since they have no voice and nothing they do will affect us.

(Malta, United Nations Library, A/CONF.151/PC/WG.III/L.8/Rev.1/Add.2, Sects. 6, 1992)^1^

This paper explores one way of empowering future generations by giving them causal efficacy over at least some members of their previous generations.^2^ Such an empowering, I argue, would be beneficial, as it contributes to diminishing a catastrophic and existential risk factor, namely the expressions of insufficient concern that some present influential individuals and institutions demonstrate towards the welfare of future people—a form of what I call ‘generational egoism’.^3^ The type of technology analysed in this paper, which would reduce the temporal parochialism of the present generation, can be characterised as a life-suspending or life-extending technology, and cryonics is one of the most well-known examples.

Although this essay focuses on the risks posed by climate change, I find it useful to deploy the broader concepts of ‘catastrophic’ and ‘existential risk’ to discuss the range of benefits related to cryonics or other relevantly similar life-suspending technologies. The reason is that such technologies may mitigate not only climate change-related risks but also risks of a broader and more heterogeneous category, namely, those risks related to some present individuals’ or collectives’ lack of (sufficient) concern for future people. More specifically, my argument includes the claim that in the case of certain catastrophic and existential risks, a general attitude—a form of generational egoism—is at least a *risk* factor, where a risk factor is something that causally increases the likelihood of a risk (see final appendix). In the case of an attitude or motivation being a risk factor, I will mean that such an attitude or motivation underlies actions increasing the likelihood of certain risks. However, under specific circumstances, some features of this attitude can become a *security* factor (i.e., something that causally decreases the likelihood of a risk). Cryonics or other similar life-suspending technology, which can give rise to such circumstances, may thus be considered a security factor for at least certain catastrophic and existential risks or risk factors. If I am right, insofar as we are interested in promoting what is beneficial to humanity, we would have a good (defeasible) reason to invest more widely in developing and making more available such technologies.

Unfortunately, at least in the academic philosophical literature, the ethical and practical impact of such technologies seems to be severely under-researched.^4^ I think that this is unfortunate and that, since life-suspending technologies would be extremely beneficial to humanity, they deserve more discussion. This paper is structured as follows. The first section briefly introduces the notions of catastrophic and existential risks. The second section of the paper clarifies what is meant by ‘cryonics’. The key aspect of cryonics relevant to this paper is that it may allow people existing at a certain time to somehow recommence or restart their lives at a later time, perhaps even significantly later (e.g., at a time significantly distant temporally from when they started the procedure). I wish to emphasise that cryonics (1) is here discussed *qua* life-suspending or life-extending technology—any other technology with the same functions would play the same theoretical role in this paper—and (2) need not be understood as a technology primarily intended to enable humans to achieve immortality. Even if we could never become immortal or everlasting, we would still have reasons to develop and make life-suspending technologies widely available. The third section outlines the general argument that cryonics is a security factor for a heterogeneous set of catastrophic and existential risks. The fourth section focuses on one of these risks: climate change. In the same section, I articulate in more detail some aspects of the problem of climate change that relate to its intergenerational character and that would be mitigated by the widespread use of cryonics. Some objections are then discussed in the final section.

## Catastrophic and Existential Risks

In this paper, I understand ‘risk’ as referring to both unwanted events with a probability of occurrence that can be determined on the basis of a priori considerations or statistical analysis, as well as unwanted events with a probability of occurrence that cannot be determined because of incomplete information, limited historical data, or an incomplete understanding of the underlying mechanisms.^5^ For the purposes of this paper, we can understand ‘catastrophic’ and ‘existential’ as referring to actual or possible bad events of significant impact in at least three dimensions: *scope* (e.g., the number of people affected), *magnitude* (e.g., the intensity of the suffering produced or level of undesirability reached), and *duration* (e.g., the amount of time they last).^6^ A catastrophic risk refers to the risk of an event having a substantial scope, magnitude, and duration. An existential risk goes further and refers to the risk of an outcome verging on the upper limit of these parameters; for instance, the permanent extinction of the whole of humanity or the imposition of an insurmountable limit on its capacity to thrive.^7^ Although I do not attempt to define the thresholds distinguishing catastrophic from non-catastrophic or catastrophic from existential risks, our intuitive judgements of various disasters equip us with a working understanding of their contours. For example, the 2010 Haiti earthquake that struck near the capital of Port-au-Prince is generally regarded as catastrophic because it caused the immediate loss of approximately 220,000–310,000 lives, considerable and lasting damage to infrastructure, the displacement of more than 1 million people, and the spread of disease. By contrast, a car accident resulting in the sudden death of one person—although certainly saddening and potentially having a devastating impact on those who loved the deceased—would normally be classified as tragic but not catastrophic for humanity. The possible extinction of all human beings owing to a giant asteroid hitting our planet, however, represents an existential risk.

A catastrophic or existential risk *factor* is something that increases the likelihood of one or more catastrophic or existential risks, whereas a catastrophic and existential security factor is something that reduces the likelihood of such risks (at least for some risks).^8^ Events or outcomes that are themselves catastrophic or existential risks may be factors for other catastrophic or existential risks. For example, climate change may directly cause catastrophic events or be responsible for increasing the likelihood of other catastrophic or existential risks—the effects of climate change may increase the likelihood of a global conflict escalating into a nuclear war, which may directly cause nuclear winter. Other examples of risk factors include the escalation of great-power competitions, the misalignment of artificial intelligence (AI), and the outbreak of engineered pandemics. Each of these scenarios, though perhaps not regarded as independently capable of resulting in, say, the extinction of humanity or its irremediable impossibility to thrive, may directly give rise to other series of events capable of (independently or in conjunction with still other events) causing such disastrous outcomes.^9^

## Life-suspending Technologies

In this paper, I understand ‘cryonics’ as referring to a technically feasible (or, at present, perhaps solely nomologically possible) life-suspending or life-extending technology, aiming to preserve a particular number of bodily parts that conjointly would be sufficient to sustain desirable features of our existence (e.g., our consciousness and personal identity), over a limited but unspecified amount of time at an extremely low temperature, in a manner that would not preclude the resumption of functionality.^10^ Ultralow temperatures can interrupt certain metabolic processes, including decomposition, and preservation at such a temperature would maintain at least some of the structures sustaining the desirable features most of us wish to preserve—for example, some of the structures of our nervous system that allow us to be (phenomenologically) conscious.

A person may desire for their whole body or parts thereof to be preserved for various reasons. For example, someone may have reached what seems, at the time, to be the natural limits of their bodily existence, yet still desire to live longer. Under the plausible presupposition that, in the future, physical life could be prolonged beyond its present apparent temporal limits—perhaps because of new technological discoveries—an individual may regard cryonics as a necessary means of satisfying their desire to continue living. Temporarily escaping such limits, however, may not be the sole or main motivation for undergoing cryonic suspension: someone may greatly desire to witness the development of a very long-term project, and witnessing the final outcome would require substantially more years than are available to a person at the time they are living (e.g., present scientists involved in the long-term project of human exploration of other planets need more than one currently normal lifetime to see their efforts come to fruition).

Although cryonics is sometimes associated with fantasies regarding immortality, presenting cryonics as being exclusively or primarily motivated by a desire to become immortal, or at least in principle to have an endless existence, can be misleading or even deleterious to a wider acceptance of and investment in this technology. In fact, the reasons for developing cryonic technologies are multiple and include several other more feasible applications compared to immortality; for example, cryonics could significantly facilitate space-travel, enable cross-temporal ‘tourism’ or migration, or satisfy intellectual curiosity to witness the further evolution of intelligent life on Earth and other planets. These projects and desires do not essentially involve any attempt to escape death indefinitely (if this were even possible).^11^

I remain neutral in this paper concerning whether signing up with any current companies offering cryopreservation or cryosuspension services would be rational in the year 2024.

## Cryonics as a Security Factor?

In this section, I first outline the connection between some catastrophic and existential risks and risk factors, and manifestations of generational egoism. I then suggest that cryonics, or any other similar life-suspending technology, can provide the conditions for countering the ill effects of such an attitude. The focus of this section is on general considerations, which I then apply to the specific case of climate change in the following section.

The main line of argument justifying the claim that cryonics can be a significant security factor for several catastrophic and existential risks starts from the point that generational egoism is, presently, a risk factor for, if not a direct cause of, certain catastrophic and existential risks. Some of these risks and risk factors are anthropogenic—highly undesirable scenarios that are or can be caused by humans (Ord, 2020, ch. 4). Some of these risks and factors are already occurring, such as climate change, global pandemics, and great-power confrontations, whereas others may loom in the near or far future, such as misaligned AI, deadly engineered pandemics, and global nuclear war. Now the magnitude, scope, and duration of some undesirable scenarios, including their associated risk factors, are partly attributable to manifestations of generational egoism, here understood as a pattern of reasoning, behaviour, and motivations (i.e., an attitude or stance) characterised by, among other things, a certain disregard or lack of appropriate concern for future generations. I use terms such as ‘self-interested’ and ‘egoism’ to broadly refer to a property of an individual or of a group being concerned exclusively for their own good or well-being. Generational egoism is characterised by the limited extension of one’s caring, such that it covers primarily or only one’s own generation or (few) presently existing people.^12^ In some people, forms of generational egoism are manifest in a lack of concern or sufficient appreciation for the value of future individuals. I will assume that manifestations of such a stance can be properly recognised in structural features of social interactions or institutions as well—for instance, I consider the second disjunct of Simon Caney’s definition of ‘Harmful Short-Termism’ (‘A government engages in Harmful Short-Termism, then, when its neglect of the future leads it to violate its responsibilities to current generations (by failing to protect their long-term entitlements) or *when it violates its responsibilities to future generations (or both)*’ [emphasis mine]) as partly being a manifestation of generational egoism at an institutional level.^13^ This definition does not imply that agents manifesting such a stance are concerned exclusively about their own individual person or group: for instance, an egoist may regard the well-being of their children or of (part of) their own generation as constitutive of their own well-being, and thus care about those other lives for selfish reasons. 14 Such a stance may be manifest in a preference for more or less significant discount rates to the value of the well-being of future people when deciding how certain resources should be distributed.^15^

Forms of generational egoism seem to partly underlie several decisions at the individual and group levels that seem to increase the likelihood of catastrophic, existential risks and related risk factors. For instance, some tobacco companies and their CEOs denied or downplayed not only the risks of smoking for existing individuals but also for future generations; in fact, they also downplayed the risks of smoking for pregnant mothers and foetuses (e.g., Philip Morris’ CEO in a notorious interview in 1971). Similarly, agents attempt to undermine scientific studies on the effects of climate change and thus contribute to exposing present and future generations to the risk of climate catastrophes.^16^ Some politicians also seem interested in short-term gains to appease their electorate and be re-elected, and thus adopt policies that are likely to discriminate against future generations.^17^ Now, I do not claim that all individuals adverse to actions aimed at, say, mitigating the ill effects of climate change, adopt the same self-interested stance. In fact, some may act in a manner that disregards the best scientific evidence and advice, and may do so for reasons not related to generational parochialism. As argued by several psychologists, a variety of more or less explicit reasons may influence individuals’ adoption of a positive or negative attitude towards actions aimed at mitigating climate change.^18^ Still, some researchers have suggested that people who hold ‘more self-transcendent and biospheric values report being more environmentally concerned, and the opposite is true for those who hold self-enhancement or egocentric values’ (Gifford & Nilsson, 2014, p. 144).^19^ For example, at an individual level, rational-egoist agents who have influence over important decisions, when pondering whether they should support certain policies that stand to benefit future people, may manifest generational egoism by not caring whether their decisions will likely cause catastrophes two or three hundred years after they or their direct descendants have all perished.

Admittedly, this sort of egoism seldom appears in an unmitigated, pure form—that is, some generational egoists may recognise *some* reasons to care about the value of distant future generations. Still, manifestations of this attitude, I believe, represent a catastrophic and existential risk factor, and several important decision-makers (e.g., politicians), influential persons, and institutions seem to display such an attitude to a sufficiently worrisome degree, at least implicitly in their actions and choices. Take, for example, certain oil companies and tycoons, dictators involved in the oil trade, their hired ‘merchants of doubts’, politicians, and so on.^20^* G*iven the positions of power that some of these egoists have, such decisions are very likely to be detrimental to the well-being of future generations (i.e., they are catastrophic and existential risk factors). As a result, some anthropogenic catastrophic and existential risks and risk factors (e.g., ineffective action to address climate change and short-term thinking) are more likely to happen partly because of preceding generations’ lack of *sufficient* concern for the well-being of all or most future people. For example, the current ineffectiveness of actions to address climate change is a clear example of a case where (i) the problem already has bad effects on some present individuals, (ii) the problem will have even worse effects in the future, both in magnitude and scope, (iii) such ineffectiveness is at least partly motivated by a lack of sufficient concern for future generations, and (iv) the problem is at least a catastrophic risk and likely also an existential risk factor.^21^ Thus, for some catastrophic and existential risks and risk factors, their likelihood is directly influenced by certain forms and degrees of generational egoism, at an individual, structural, or institutional level.^22^

My next general claim in this section is that cryonics can be regarded as a security factor because, among other things, it would make it rational for even some generational egoists to expand their circle of care to future people—or, at least, to increase their level of care for the well-being of future generations. The reasoning supporting this claim is relatively straightforward: because the generational egoist who would undergo cryonic preservation may wish to (1) live at some future time, including when the worst effects of some of their otherwise egoistic choices will have already occurred or will be imminent, and (2) obtain advantages from future people, (3) it would thus be rational for them to (a) act in a manner that safeguards the survival and flourishing of humanity; (b) act in a manner that they would expect future generations to reciprocate; or, at least, (c) promote policies, attitudes, or structural changes aimed at decreasing long-term catastrophic or existential risks. If it is rational for anyone to behave in a manner that demonstrates concern for people who may benefit them in the present, it is also rational for them to demonstrate sufficient concern for those whom egoists expect will benefit them in the future. It may be argued that, for cryonics to become a sufficiently effective security factor, it ought not only to influence particular generational egoists but also to affect structural and institutional factors.^23^ This claim is compatible with what I have suggested. In general, the structure of my argument can be adapted to the recognition that failure to tackle certain catastrophic and existential risks may be related to structural factors: if the use of cryonics or other life-suspending technologies were to become structural factors of our institutions, then their presence would presumably mitigate manifestations of generational egoism that appear at those levels as well.^24^ For instance, in a society in which cryonics were widely accepted, harmful political short-termism may more likely appear as one of the several myopic prejudices that have affected us in the past. This point is related to the prudential stance mentioned above—as it would not be rational to act in ways showing a racist attitude where one’s life would depend on people of another race, similarly it would not be rational to display generational egoism when one’s future existence depends on future generations. Being myopic about the well-being of future generations may become irrational at an individual and institutional level. In the following section, I expand the application of the previous line of reasoning to the case of anthropogenic climate change.

## Cryonics as a Security Factor: The Case of Climate Change

To begin, what are some of the general features of climate change that make it difficult to manage or solve? Given the complexity of the problem and the number of different agents and institutions involved, it seems wrong to reduce all the difficulties in addressing climate change to a unifying or comprehensive principle or factor that might explain all the relevant agents’ explicit and implicit motivations to pursue ineffective courses of action or to hinder others’ actions to reduce the ill effects of climate change (Gifford & Nilsson, 2014; Jamieson, 2014, ch. 3). For instance, to say that the problem of addressing climate change is attributable exclusively to the selfishness of a certain generation of agents would fail to capture all the subtleties of the relevant agents’ motivations and irrationalities. In fact, as already mentioned, we may assume that some people are truly convinced that anthropogenic climate change is a conspiracy and does not pose any real risk to present or future people. At least in some cases, these deniers would be likely to promote effective climate action were they better informed or better reasoners. So, I do not claim that one or all the principles or causes listed in this section capture all the risk factors of climate change related to human agency. Rather, my claim connecting climate change and cryonics is a rather modest one: cryonics (or other similar life-suspending technologies) is a security factor to the extent that it is likely to mitigate *some* important risk factors of climate change related to influential human agents or institutions. What, then, are some of the risk factors of climate change that cryonics may mitigate?

My answer to this question largely draws from Stephen Gardiner’s perfect moral storm (PMS) analysis (Gardiner, 2011).^25^ In brief, Gardiner argues that anthropogenic climate change has structural features that converge to greatly obstruct our capacity to make ‘the hard choices’ required to effectively address it. Among these structural features, Gardiner includes the intergenerational aspect of the problem. In turn, this intergenerational aspect has several key structural elements that contribute to the ‘perfect moral storm’. For instance, the causes and effects of climate change are spread across time: some of the worst effects are set to occur years after their cause—for example, the effects of emissions of carbon dioxide into the atmosphere are cumulative, and their worst effects are likely to be felt years after their emission. This delay leads to a deferred experience of some present polluting actions; however, the polluting activities of each generation accumulate, and not all of the most adverse consequences of such polluting are borne by the same generation responsible for the pollution. According to Gardiner, this backloading and deferral generate what he calls ‘an intergenerational buck-passing problem’: because most of the negative consequences of generation G_1_’s polluting activities will be felt mostly by the successive G_2_, G_1_ may be incentivised to ‘pass on the bill’ to reduce emissions to any later G_i_, and thus continue over polluting.^26^ This lack of incentive can be seen as involving elements of generational egoism. If the context and incentive structure remain the same, G_2_ would act in the same manner, as would all subsequent generations, thereby exacerbating the situation until one subsequent generation lacks the capacity to pollute further or perhaps until an imminent and likely inevitable catastrophe occurs.

A further obstacle to remedying anthropogenic climate change is what Gardiner calls ‘the problem of interaction’. In various models of rationality used to discuss this problem, the issue is the lack of sufficient benefits accruing from direct, reciprocal interactions between far away generations. In short, future generations seem to have little to offer prior generations comparable to what, say, interactions between groups of individuals in the same or overlapping generations can offer. Now, problems of conflicting interests may be ameliorated by repeated and mutually beneficial interactions between relevant agents (Axelrod, 1984). Unfortunately, these solutions do not seem to be presently available in the case of present–future generational interactions involving polluting activities, because the relevant agents are unable to appeal to a context of mutual beneficial interactions, nor can they appeal to some direct forms of reciprocity.^27^ In fact, Gardiner holds that neither ‘repeated interaction’ nor (apparent) ‘mutual benefit’ are features of the interactions between present and future generations (Gardiner, 2011, p. 37). The problem of a lack of repeated and mutually beneficial interactions seems to derive from the causal asymmetry in which different generations stand, which in turn is primarily caused by the different temporal locations of past, present, and future people. To sum up, some of the difficulties of forging consensus solutions to the problem of climate change include: (a) the temporal dispersion of the effects of climate change, (b) the lack of interaction between different generations, (c) the lack of ‘repeated interactions’ between and among the agents concerned, and (d) the lack of ‘mutual benefit’ among the agents concerned. Any factor that would properly remove or make some (or all) of these important factors less problematic or likely to occur would count as a security factor.

For individuals of any generation choosing a relevant life-suspending technology, cryonics (or other similar life-suspending technologies) would enable the following:

1. a fuller experience of the ill effects of their polluting activities;
2. interactions between generations (understood as age cohorts);
3. the establishment of relationships of mutual benefit across generations;
4. repeated interactions across generations (if cryonics were a repeatable procedure).

Let us explore in more detail each of the previous points to better elucidate in what sense cryonics would be a security factor. In relation to (1), the problem of dispersed emissions (and thus the temptation of intergenerational buck-passing) would not be directly mitigated by cryonics making emissions contemporaneous; rather, for those utilising a life-suspending technology to suspend their lives for a sufficient amount of time, the temporality of emissions would make it likely that they would experience the delayed effects of their polluting activities. Thus, they would be personally invested in the matter. Someone may object that making cryonics more widespread would require additional energy and thus would create more pollution. This possible objection does not strike me as being particularly strong, at least at the level of generality that characterises this paper. For instance, we may reply that life-suspending technologies, similar to life-saving technologies, should be prioritised over other comparatively less important technologies. The additional energy required to make cryonics more widespread should be taken from other industries. Besides, making cryonics more widespread may further incentivise the development and use of clean energy, partly because people interested in living in the future would presumably like to live in a clean environment. Regarding (2), cryonics would make it possible to establish direct causal relations between and among individuals who would not otherwise be in such mutual causal relationships. People taking advantage of a life-suspending technology would causally depend on the actions of future individuals for being ‘awakened’—how far in the future these individuals temporally reside would clearly depend on the specifics of the life-suspending technology and on other social, individual, and environmental conditions. Regarding (3), people interested in being suspended would presumably expect to receive a benefit from future (in relation to their temporal location) individuals. Such an expectation would be based also on their prior, effective, and perhaps publicly performed beneficial-to-future-generations behaviour, thus ameliorating aspects of their possible generational egoism. This last point is related to (4): supposing that suspending one’s life can be a repeatable procedure, establishing repeated causal connections across multiple generations would be possible. People who cooperated intergenerationally because they wished to receive the benefit of being ‘reactivated’ in the future would have an incentive to further collaborate with several other future generations, were they interested in repeating the procedure. Connections of direct mutually beneficial cooperation would thus open new and interesting scenarios involving multigenerational collaboration, and they may even lay the foundation for a multi-generational human community. Such a community would provide the ideal theoretical and practical base for grounding substantial claims of obligations and justice towards all members of such a community, in a manner that would not discriminate their accidental temporal location.^28^

As mentioned at the end of the previous section, everyone interested in undergoing repeated cryonic procedures would thus have reasons to incentivise the dismissal of generational egoism and not to just simulate compliance with a code of behaviour conducive to reducing the risk of climate change. They would presumably tend to promote the internalisation of measures and dispositions that would ensure a future not plagued by the ill effects of climate change, assuming that such egoists are not misinformed. Besides, if the egoists believed that their actions were making no difference, they would have reason to ensure that at least some of their actions would start making some difference and incentivise structural change in their society. For instance, they would tend to vote or show public support for candidates endorsing policies in favour of tackling climate change. In addition, individuals expecting to be revived by future generations would be incentivised to demonstrate their social value and their positive impact on the preservation of the environment—or at least to attempt to ensure that certain desirable goods are preserved for future generations. After all, they may reason, in possible conditions of scarcity, why would future generations be otherwise incentivised to revive them? Although it may well be the case that a specific future generation, composed of specific individuals, would not have existed were it not for, say, the reckless actions of a previous one, this would hardly ground a significant degree of gratitude or compassion on the side of the affected future people.^29^ Besides, future people may question the rationality of reviving individuals whose behaviour and attitudes were so reckless towards climate change in the past, if not to punish them for what they were not punished for when they were alive.

In general, cryonics would open the theoretical and practical possibility of having interactions across generations in the form of direct mutual benefits—after all, present generations interested in cryonics would benefit from being revived by future generations. At the same time, future generations would benefit from the change of attitude in members of their previous generations. Provided that cryonic procedures can be successfully implemented several times successively on the same individuals, individuals existing at any time would see future generations as possible partners in mutually advantageous and *repeatable* interactions. In the case of well-informed individuals, such advantageous interactions would include actions aimed at reducing risks attributable to climate change. All those interested in undergoing life-suspending procedures, including egoists and generational egoists, would thus have additional reasons to ensure the success of policies aimed at combating the ill effects of climate change.

## Conclusions

Several objections can be raised, and have been raised, against cryonics in general. I do not have the competence to address the technical ones, so I refrain from discussing them here. Suffice to say, the objections to the current state of the technology or its future developments do not seem to invoke a nomological impossibility to any type of life-suspending procedures. The main points and arguments of this paper would still stand insofar as it is nomologically and technologically possible to devise life-suspending or life-extending technologies that would allow several successive generations to be ‘contemporaries’ (at least for some time).

It is difficult to speculate what the degree of cultural and social acceptance may turn out to be in case life-suspending technologies become more widely available. Still, given the structural problems of, say, climate change, it is important to promote those factors contributing to the creation of a cross-temporal community—one that could involve mutually advantageous exchanges to help ameliorate the short-sightedness of at least some influential decision-makers and institutions. It may be argued that there are substantial worries related at least to the beginning of such an intergenerational community if it started with, for example, tech billionaires or other powerful individuals. Would these individuals still have incentives to tackle only problems that worried them alone, or would they have to worry about the possibility of returning to life deprived of their excessive wealth and power? Exclusively for the argumentative purposes of this paper, this may not necessarily be an insurmountable problem. For instance, in the case of climate change, I suspect that even tech billionaires (unless they are badly informed) would want to incentivise those policies that would make the environment liveable for when they are revived. Although some may indeed be preparing for an environmental catastrophe by building secret compounds or refuges (Rushkoff, 2023), it is unlikely that they would have a better revived life exclusively in a sealed bunker or islands rather than having the whole world as a playground (as they do now), with all its related resources. Besides, there is a substantial element of uncertainty in predicting the future structure of societies; as a result, powerful people in the present may also want to ‘play safe’ and attempt to openly try to decrease the chances of catastrophic risks—not to mention existential ones—by combating generational egoism. After all, at least presently, it is unclear whether they would be allowed to retain their wealth, if only because the legal status of a ‘revived’ person is unclear, or because of other societal changes. For these reasons, also at an initial stage, the possible benefits of cryonics seem to outweigh its possible side effects deriving from possible abuses.

More discussion on cryonics as a security factor is certainly required, but I think that the arguments presented in this paper can reasonably motivate scholars to further investigate the range of benefits that cryonics, or any other life-suspending technologies, can provide against various risks related to humanity’s tendency towards short-term thinking and decision-making at different levels.

## Appendix 1. Risk and Security Factor

Following Ord (2020, p. 178), we can adopt the language of probability theory to make the notions of a risk and of a security factor more precise:

- –Where R is an event (e.g., a great-power war);
- –f is the quantitative measure of the likelihood of R (e.g., the probability of the occurrence of such a war or the probability of cryonics becoming an accessible technology);
- –f_min_, f_sqt_, and f_max_ are the minimum achievable value of f, the status quo with respect to a certain time t, and the maximum achievable value, respectively;
- –C and E are the occurrence of particular catastrophic and existential outcomes, respectively,

The difference between Pr (C|f = f_sqt_) and (Pr (C|f = f_min_) can be taken as the contribution that R makes to a catastrophic risk and Pr (E|f = f_sqt_) and (Pr (E|f = f_min_) as the contribution that R makes to an existential risk. These equations represent the extent to which total C or E would be lowered if we eliminated R.

The difference between Pr (C|f = f_sqt_) and (Pr (C|f = f_max_) refers to the potential of R to cause C, and that between Pr (E|f = f_sqt_) and (Pr (E|f = f_max_) refers to the potential of R to cause E. These equations represent how much catastrophic and existential risk could arise if the risk factor in question were to worsen or be introduced.

These definitions can be understood as indicating that, if introducing, exacerbating (when R is present), or not eliminating R increases the likelihood of C or E happening, then R is a risk factor with respect to C or E. Conversely, if introducing, improving (when R is present), or not eliminating R decreases the likelihood of C or E happening, then R is a security factor with respect to C or E. Clearly, R may be a risk factor with respect to some Cs or Es and, simultaneously, be a security factor with respect to other Cs or Es. Another way in which R can be a catastrophic or existential risk or security factor is by affecting other catastrophic or existential risks or security factors. For instance, a catastrophic risk factor may affect the likelihood of the occurrence of some other catastrophic risk factor.

## Footnotes

1 Cited in Caney (2022, p. 143). See http://193.166.3.2/pub/doc/world/UnitedNations/EnvironConf/PreConfDocs/wg3l8add02. (Sect. 6).

2 Alex Gosseries understands ‘generations’ as referring to birth cohorts, defined as ‘sets of individuals born during a certain period’ (Gosseries, 2001, 2023). Other interpretations presuppose an understanding of ‘future generations’ according to which their distinctive feature is that we cannot realistically anticipate having a shared existence with them (Golding, 1972, p. 86). Other authors use ‘future generations’ simply to refer to those future people who do not yet exist at the time of speaking. In this paper I follow the latter usage, unless an alternative usage is specified. Gardiner (2011, pp. 145–148), Caney (2018a, b) for additional discussion.

3 In this paper, I do not directly address the issue of whether we have duties towards future people. See Mulgan (2006), Meyer (2021), and Gosseries (2023) for discussion.

4 As of April 2024, there are only 14 entries (including unpublished dissertations) under the category ‘cryonics’ in Philpapers. See https://philpapers.org/browse/cryonics.

5 See Buchak (2014, p. 8) and Peschard et al. (2023, ch. 1) for ‘risk’.

6 See Bostrom (2003, 2013, 2014) and Ord (2020) for the terminology of existential risk. See Avin et al. (2018) and Cotton-Barratt et al. (2020) for classifications of catastrophic and existential threats. See Stefansson (2020) for the meaning of ‘catastrophic’.

7 The formulation of a precise account of what the flourishing or thriving of humanity is goes beyond the purposes of this paper. According to Desmond and Ramsey (2023), such a flourishing implies humanity’s achieving some kind of success.

8 See the appendix for further details. I assume that the increase in likelihood between events discussed in the text is due to relevant causal connections.

9 See Appendix 1 for a more rigorous definition of a risk and security factor.

10 See Shaw (2009, 2013), Minerva (2018, p. 8), Moen (2015), Sandberg and Minerva (2015), and Sauchelli (2018, pp. 185–188) for some philosophical work on the topic.

11 Philosophical arguments against the rationality or desirability of immortality (e.g., the notorious Makropulos case described by Bernard Williams) are thus orthogonal to the desirability of undergoing a cryonic procedure. For this reason, I do not discuss them. See Fischer (2012) on immortality and Overall (2011) for a position on lifespan extension with which I am largely sympathetic.

12 Contrast this stance with the attitude of love for humanity as described in Scheffler (2013, 2018).

13 Caney (2019).

14 See Shaver (1998) for an introduction to egoism and Hooker (2013) for a discussion of partiality and impartiality. I remain neutral with respect to what constitutes the well-being of individuals.

15 See Broome (2012, ch. 8), Cowen and Parfit (1992), Greaves (2017), Schelling (2000), and Zuber and Fleurbaey (forthcoming) for critical discussions of discount rates.

16 Among the multiple sources of evidence concerning the case of the tobacco industry, see Karagueuzian et al. (2012), and for a study of the case of climate change deniers and the tobacco industry, see Conway and Oreskes (2010).

17 Caney (2022) for additional discussion.

18 See Gifford (2011), Gifford and Nilsson (2014), and Knez (2016). See also Jamieson (2014, ch. 3) for relevant considerations.

19 See, for instance, Milfont and Gouveia (2006), Schultz and Zelezny (1999) and Stern (2000). Stern (2000, p. 412) asserts, ‘Empirically, measures of egoistic values have been negatively correlated with indicators of environmentalism’. I take it that many of their indicators for egoistic attitudes are manifestations of what I have called an egoistic stance.

20 See Conway and Oreskes (2010) and Wenar (2016). One recent example is Gazprom’s drilling successes in the Arctic region; on this matter, see Afanasiev (2022).

21 Gardiner (2011) and Jamieson (2014) make similar points.

22 I do not mean that all instances of partiality favouring presently existing individuals are unjustified. In some cases, requirements of justice or other considerations may compel the choice that certain resources ought to be distributed in a manner that demonstrates a certain level of partiality to presently existing people (Broome, 2012).

23 See, for instance, Malm (2016). Thanks to an anonymous referee for this point.

24 Presumably, those individuals choosing to utilise life-suspending technologies would tend to incentivise or promote such structural changes.

25 Other important works include Broome (2012), Jamieson (2014), Kopec (2017), McKinnon (2012), Moellendorf (2014), Page (2006), and Shue (2014). My main sources for all technical and scientific evidence are IPCC (2022) and IPCC (2023).

26 Gardiner (2011, p. 35). See Hourdequin (2022) for a relevant discussion.

27 Similar problems have been raised in the context of contractualist accounts of intergenerational justice. See Arrhenius (1999), Heath (2013), and Kumar (2021).

28 See de-Shalit (1995) for an early outline of a ‘transgenerational community’ and some applications of the theory. Avner de-Shalit does not consider the possibility of realising such a community by technological means, nor does he consider a human (i.e., inclusive of all existing and future people) community. On his view, a transgenerational community would persist through time in virtue of its cultural and moral connections. On my account, this condition would be strengthened by the actual persistence of the individuals composing such a community.

29 This interesting application of the notorious non-identity problem has been brought to my attention by an anonymous referee.
